# Delayed repair of the facial nerve and its negative impacts on nerve and muscle regeneration

**DOI:** 10.1590/1678-9199-JVATITD-2023-0093

**Published:** 2024-05-27

**Authors:** Cleuber Rodrigo de Souza Bueno, Daniela Vieira Buchaim, Benedito Barraviera, Rui Seabra Ferreira, Paulo Sérgio da Silva Santos, Carlos Henrique Bertoni Reis, Marcelo Augusto Cini, Milton Carlos Kuga, Geraldo Marco Rosa, Rogerio Leone Buchaim

**Affiliations:** 1 Department of Biological Sciences, Bauru School of Dentistry (FOB), University of São Paulo (USP), Bauru, SP, Brazil.; 2 Dentistry School, University Center of Adamantina (UNIFAI), Adamantina, SP, Brazil.; 3 Medical School, University Center of Adamantina (UNIFAI), Adamantina, SP, Brazil.; 4 Graduate Program in Structural and Functional Interactions in Rehabilitation, University of Marilia (UNIMAR), Marília, SP, Brazil.; 5 Graduate Program in Anatomy of Domestic and Wild Animals, Faculty of Veterinary Medicine and Animal Science (FMVZ), University of São Paulo (USP), São Paulo, SP, Brazil.; 6 Center for the Study of Venoms and Venomous Animals (CEVAP), São Paulo State University (UNESP), Botucatu, SP, Brazil.; 7 Graduate Program in Tropical Diseases, Botucatu Medical School (FMB), São Paulo State University (UNESP), Botucatu, SP, Brazil.; 8 Department of Surgery, Stomatology, Pathology and Radiology, Bauru School of Dentistry (FOB), University of São Paulo (USP), Bauru, SP, Brazil.; 9 UNIMAR Beneficent Hospital (HBU), University of Marilia (UNIMAR), Marília, SP, Brazil.; 10 Medical School, University of West Paulista (UNOESTE), Guarujá, SP, Brazil.; 11 Department of Restorative Dentistry, School of Dentistry, São Paulo State University (UNESP), Araraquara, SP, Brazil.; 12 Dentistry School, Faculty of the Midwest Paulista (FACOP), Piratininga, SP, Brazil.

**Keywords:** Facial nerve, Nerve regeneration, Photobiomodulation, Fibrin sealant, Biopolymers

## Abstract

**Background:**

In this experimental protocol, we evaluated the immediate and delayed repair of the buccal branch of the facial nerve (BBFN) with heterologous fibrin biopolymer (HFB) as a coaptation medium and the use of photobiomodulation (PBM), performing functional and histomorphometric analysis of the BBFN and perioral muscles.

**Methods:**

Twenty-eight rats were divided into eight groups using the BBFN bilaterally (the left nerve was used for PBM), namely: G1 - control group, right BBFN (without injury); G2 - control group, left BBFN (without injury + PBM); G3 - Denervated right BBFN (neurotmesis); G4 - Denervated left BBFN (neurotmesis + PBM); G5 - Immediate repair of right BBFN (neurotmesis + HFB); G6 - Immediate repair of left BBFN (neurotmesis + HFB + PBM); G7 - Delayed repair of right BBFN (neurotmesis + HFB); G8 - Delayed repair of left BBFN (neurotmesis + HFB + PBM). Delayed repair occurred after two weeks of denervation. All animals were sacrificed after six weeks postoperatively.

**Results:**

In the parameters of the BBFN, we observed inferior results in the groups with delayed repair, in relation to the groups with immediate repair, with a significant difference (*p* < 0.05) in the diameter of the nerve fiber, the axon, and the thickness of the myelin sheath of the group with immediate repair with PBM compared to the other experimental groups. In measuring the muscle fiber area, groups G7 (826.4 ± 69.90) and G8 (836.7 ± 96.44) were similar to G5 (882.8 ± 70.51). In the functional analysis, the G7 (4.10 ± 0.07) and G8 (4.12 ± 0.08) groups presented normal parameters.

**Conclusion:**

We demonstrated that delayed repair of BBFN is possible with HFB, but with worse results compared to immediate repair, and that PBM has a positive influence on nerve regeneration results in immediate repair.

## Background

Peripheral nerve injuries are common and despite advances in treatments, they still have limitations in the complete restoration of motor and sensory functions [1-11]. Among the cranial nerves, the seventh nerve pair (facial-intermediate), when it is injured, loss of facial expression occurs, leading to functional and aesthetic loss of the face, affecting the ability of individuals to socialize [12-15]. The population involved is young, which causes significant socioeconomic damage to society [16].

The lesion classified as neurotmesis is considered to be the most serious, as it causes complete loss of nerve continuity [17], representing a major challenge in peripheral nerve repair methods [15, 18, 19]. To allow nerve regeneration through axonal sprouting, surgical treatment for repair is suggested [18, 20]. The gold standard for repairing this type of injury is immediate end-to-end neurorrhaphy with suture [21-23]. However, limitations of this type of treatment are evident, highlighting the difficulty of the technique and the inflammatory and infectious processes that can occur, leading to failures and limitations in the treatment [24, 25]. Therefore, new methods have been tested to improve functional and morphological results.

The heterologous fibrin biopolymer (HFB) has been tested for several clinical situations, among them, as a surgical glue for repairing peripheral nerves. The bioproduct is purified and extracted from the venom of the *Crotalus durissus terrificus* snake, and has been shown to be biocompatible, biodegradable, hemostatic, and does not produce adverse reactions. It was conceived and manufactured by the Center for the Study of Venoms and Venomous Animals (CEVAP/Unesp, Botucatu, Brazil) [26-28]. In previous studies of this group, HFB was used for the coaptation of nerve injuries, showing promising results similar to sutures [29-31]. However, there is still no study with the coaptation of neurotmesis with the heterologous fibrin biopolymer (HFB) in peripheral nerves analyzing the results in delayed repairs, and with the study of the possible alterations in the fibers of the facial muscles [32-34].

Another aspect that influences the prognosis of regeneration is the waiting time for surgical repair, as there are positive effects on repair immediately or with a short period of injury. However, there are situations in which there is a delay in diagnosing the injury [35-38] or traumatic etiologies, in which the patient must be stabilized first, and then nerve repair is performed; therefore, the period of delay in repair is inevitable. In a study that evaluated the etiologies of facial nerve injuries [16], it was revealed that approximately 90% of injuries occurred due to car accidents, which often lead to delayed nerve repair.

In this context, the time delay for the repair and the consequent non-communication of the proximal stump (which has the Schwann cells responsible for the regenerative process) with the distal stump, will cause structural and molecular changes in this stump, decreasing the functional recovery of the nerve. In addition, the maintenance of muscle tissue is dependent on the nervous and neurotrophic stimulus of the injured nerve, which is lost, resulting in an atrophic process in muscle cells, and consequent invasion of connective tissue between muscle compartments, with less power of nerve interaction with the motor endplate. The reported alterations are progressive and advanced, and worsen the prognosis with the time of denervation [39]. The described processes lead to a slower and poorer functional recovery in quality, due to the difficulty of the new neuromuscular interaction.

One of the complementary methods that have been tested to help in a faster and more efficient recovery of the peripheral nerves is photobiomodulation (PBM), using low-level laser therapy (LLLT), being a viable, non-invasive, and safe option. Effects such as increased metabolic rate, increased mitochondrial activity [40-43], and greater transport of substances, including oxygen, increase the activation of cell transcription factors, proliferation, survival, tissue repair, and nerve regeneration [44, 45]. These benefits affect the neuromuscular recovery of injured nerves, such as the decrease in the muscle degeneration process [46-49]. However, there is still no consensus protocol for its use, with few studies using PBM in facial nerves. Our group previously used a photobiomodulation protocol with LLLT in the repair process of peripheral nerve defects, obtaining promising results [30, 31, 45, 50].

Preliminarily, we carried out the study with immediate repair of the buccal branch of the facial nerve using HFB and PBM, obtaining promising results [51]. Thus, aiming to translate this method to an important clinical situation in facial nerve injuries, in which nerve repair is performed late, this study describes in an unprecedented way the effects of using HFB as a means of coaptation for the repair of delayed neurotmesis associated with the use of laser therapy. Therefore, the objective of this study was to analyze the peripheral effects of immediate and delayed repair of the lesion of the buccal branch of the facial nerve (BBFN), repaired with fibrin biopolymer associated or not with photobiomodulation.

## Methods

### Experimental design

Twenty-eight male Wistar rats (*Rattus norvegicus*) were used. The animals were 90 days old, weighing approximately 250-300 g at baseline. All animals were kept in appropriate boxes, received water, and fed *ad libitum*, with no restrictions on movement, respecting the 12-hour light/dark regime and an approximate temperature of 22 ºC. Throughout the experimental period, signs and symptoms of stress and unusual behavior of the animals were observed. The study was carried out according to the ARRIVE protocol (Animal Research: Report of in vivo Experiments) and based on the principles of the NC3Rs (National Center for Replacement, Refinement, and Reduction of Research Animals). It was also carried out in accordance with the guidelines of the Declaration of Helsinki and was approved by the Ethics Committee on the Use of Animals of the University of Marília (UNIMAR, Marília, Brazil) with protocol 033/2020; November 13, 2020). 

The experiment was carried out with the buccal branch of the facial nerve (BBFN) on the right and left of all animals. The preclinical protocol was to perform photobiomodulation therapy (PBM) on the left side with the proposed protocol, and on the right side of the face without PBM. Groups G1 to G6 were euthanized six weeks after the experimental surgery, and groups G7 and G8 were euthanized four weeks after surgery (delayed repair).

**Figure 1.**
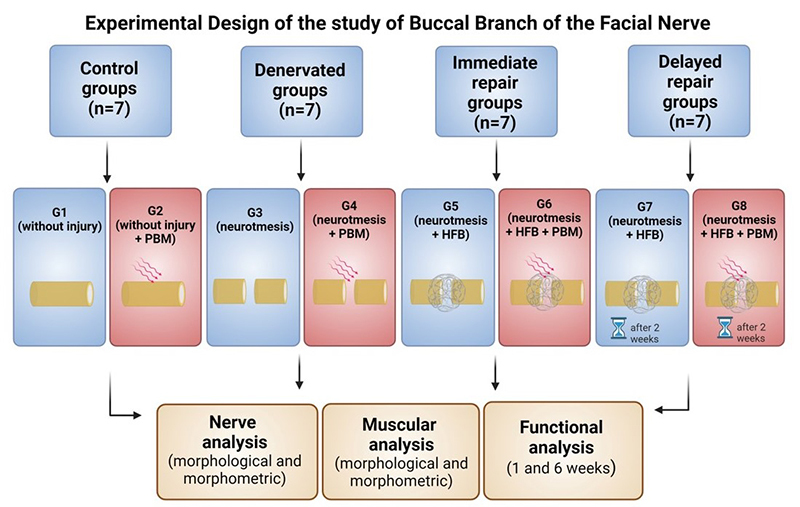
Experimental model of the study with the following groups: control (G1 and G2), in which we analyzed the intact buccal branch of the facial nerve (BBFN); denervated (G3 and G4), who underwent a surgical procedure to prevent new spontaneous reinnervation; experimental groups (G5-G8), where the total section of the nerve and the coaptation with the heterologous fibrin biopolymer (HFB) were carried out immediately (G5 and G6) and delayed (G7 and G8). After the experimental period, the animals were euthanized, and morphological, morphometric, and functional evaluations were performed. In groups G1, G3, G5 and G7: right BBFN; in groups G2, G4, G6 and G8: left BBFN.

The animals were randomly divided into eight groups: control groups (G1 + G2; n = 7), denervated groups (G3 + G4; n = 7), immediate repair groups (G5 + G6; n = 7), and delayed repair groups (G7 + G8; n = 7). In detail, according to the side of the face on which the injury occurred and its respective treatments: G1: Control group, right BBFN (without injury); G2: Control group, left BBFN (without injury + PBM); G3: Denervated right BBFN (neurotmesis); G4: Denervated left BBFN (neurotmesis + PBM); G5: Immediate repair of right BBFN (neurotmesis + HFB); G6: Immediate repair of left BBFN (neurotmesis + HFB + PBM); G7: Delayed repair of right BBFN (neurotmesis + HFB); G8: Delayed repair of left BBFN (neurotmesis + HFB + PBM) (Figure 1).

### Heterologous fibrin biopolymer (HFB)

The constituents of the heterologous fibrin biopolymer, its formula, and its forms of application are by patent number BR 102014011432-7, issued on July 6, 2022, by the National Institute of Industrial Property of Brazil (INPI). This bioproduct underwent a clinical trial for the treatment of venous ulcers phase I/II [52], which proved its efficacy and safety for therapeutic use in humans, standing out as a promising translational potential in regenerative and therapeutic medicine.

In the groups with neurotmesis repair (G5-G8), HFB was used. This material was kindly provided by the Center for the Study of Venoms and Venomous Animals (CEVAP) of the São Paulo State University (UNESP, Botucatu, Brazil). HFB has three components that have been removed from the freezer, thawed, and mixed before use. In sequence, with the aid of a micropipette, the substances were applied for the coaptation of the stumps of the injured nerve. First was the thrombin-like enzyme fraction, fraction 1 (5 µL), the second contained calcium chloride diluent (5 µL), and the last (fraction 2) fibrinogen extracted from buffalo blood (10 µL) *Bubalus bubalis*. To certify its adhesion, after one minute of application and polymerization of the HFB, slight traction of the coapted nerve stumps was performed.

### Surgical procedures

For all surgical procedures, the animals underwent general anesthesia with intramuscular injection of tiletamine hydrochloride and zolazepam hydrochloride (10 mg/kg, Telazol^®^, Fort Dodge Laboratories, IA, USA). Trichotomy was performed with the aid of a hair trimmer (Philips^®^ Multigroom QG3250, São Paulo, Brazil) in the region of the bilateral face of the animals along the labial commissure to the tragus, in order to obtain a smooth and hairless surface. Next, the animal was positioned in lateral decubitus on a surgical table and antisepsis was applied with 10% polyvinylpyrrolidone-iodine (PVPI, Povidine^®^ Antiseptic, Vic Pharma, São Paulo, Brazil).

### Denervation surgery (G3-G8 groups)

Using a surgical microscope (DF Vasconcelos^®^, São Paulo, Brazil) a preauricular incision was made with a number 15 scalpel blade (Embramax^®^, São Paulo, Brazil) measuring approximately 3 cm. Then there was the divulsion by planes, identification of the platysma muscle superficially, and its sectioning. The BBFN was recognized, released, and sectioned in its central portion (midpoint of the line from the tragus to the labial commissure in lateral view). In order to avoid spontaneous regeneration, the proximal stump was manipulated at 180**º** degrees and sutured to the adjacent muscle fascia, while the distal stump was manipulated at 180**º** degrees and sutured to the adjacent musculature [33, 53, 54]. Finally, the skin was sutured with a simple stitch using 4-0 nylon thread (Ethicon^®^, Johnson & Johnson, São Paulo, Brazil).

### End-to-end neurorrhaphy with HFB (G5-G8 groups)

After following the same steps as the previous protocol, and its work of recognition and release of the BBFN, sectioning (neurotmesis) was carried out in its central portion (point from the center of the tragus line to the labial commissure in lateral view), and anatomical approximation of the stumps of the nerve, with coaptation with HFB in animals from G5 and G6. In the groups with delayed repair (G7 and G8), the same surgical procedures were performed for neurotmesis, but the new surgery for coaptation with HFB was performed two weeks after nerve injury [30, 54]. The skin suture completed the surgical phase with a simple stitch using 4-0 Ethicon^®^ nylon thread (Johnson & Johnson, São Paulo, Brazil).

### Post-surgical care

After the surgical procedures were completed, all animals received a single dose of the antibiotic Flotril^®^ 2.5% (Schering-Plough, Rio de Janeiro, Brazil), at a dosage of 0.2 mL/kg and dipyrone analgesic Analgex V^®^ (Agener União, São Paulo, Brazil) at a dose of 0.06 ml/kg in intramuscular applications. The injectable analgesic was maintained for three days, in addition to the use of analgesic drops Paracetamol^®^ (Generic medication, Medley, São Paulo, Brazil) at a dose of 200 mg/kg, 6 drops/animal dissolved in the water available in the drinker until the period of euthanasia.

### Photobiomodulation protocol (PBM)

Treatment with PBM began in the immediate postoperative period and continued for five weeks, with a single weekly application, always occurring on the same day of the week throughout the protocol. In animals from G7 and G8 (delayed repair), PBM applications were performed after neurorrhaphy, totaling four weeks of treatment.

The animals were manually immobilized (mild restraint) and their sedation was not necessary during PBM. Prior to LLLT applications, the device was calibrated and tested to certify the dose. The application was performed in three points, the first point at the section site (neurotmesis), the second point 10 mm anterior to the section (distal stump), and the third 10 mm posterior to the section (proximal stump), in light contact with the skin. All study groups received the PBM protocol on the BBFN on the left side using the low-level laser Gallium Aluminum Arsenide (GaAlAs), Therapy XT DMC^®^ (São Carlos, Brazil), following the parameters in Table 1. 

**Table 1.** PBM protocol.

Parameter	Unit/Description
Type of laser	GaAlAs/infra-red
Output power	100 mW ± 20%
Wavelength	808 nm ± 10 nm
Power density	2.32 W/cm^2^
Energy density	93.02 J/cm^2^
Beam area	0.043 cm^2^
Fiber diameter	2.35 mm
Fiber area	0.04337 cm^2^
Total power	12 J
Beam type	Positioned perpendicular to the skin
Emission mode	Continuous
Form of application	Three points in nerve injury
Irradiation duration	40 s per point
Irradiation time of each application	120 s
Treatment time	Immediately after surgery and once a week until euthanasia

GaAlAs: gallium aluminum arsenide; mW: milliwatts; nm: nanometers; W: watts; J: joules; cm^2^: square centimeters; s: seconds.

### Functional analysis of rat vibrissae

During periods of one to six weeks postoperatively, observations of the animals' vibrissae were made. The rats were placed in a box with a black background and observed both during their spontaneous movements and also in response to the researcher's stimuli (3 to 4 clapping of hands). Observations were performed blindly, without the evaluator knowing which group was being evaluated. Scores were attributed following the methodology of de Faria et al. [54]. 

### Sample collection and euthanasia

Six weeks after the experimental surgery, the animals were anesthetized, the BBFN was carefully dissected and 10 mm of the stump distal to the neurotmesis was collected in the experimental groups (G5-G8), denervated (G3 and G4) and the intact nerve in the control group (G1 and G2), under 16x magnified view of the surgical microscope (DF Vasconcelos^®^, São Paulo, Brazil). Next, the muscles of facial expression in the perioral region of all groups were dissected and carefully removed. Euthanasia was performed in a silent environment and away from other animals, using an anesthetic overdose (triple dose - 240 mg/kg tiletamine hydrochloride + 30 mg/kg zolazepam hydrochloride).

### Histological and morphometric processing of nerve and muscle

The samples collected from the nerves were fixed in a 10% buffered formaldehyde solution for 24 h and the HistorResin protocol was performed (Leica Mycrosistems^®^, Wetzlar, Germany) [31]. Sections were performed using a semiautomatic microtome (Model RM2245, Leica Microsystems^®^, Wetzlar, Germany) with a thickness of 5 µm. The slides were stained with osmium tetroxide and counterstained with 1% Toluidine Blue in distilled water. The sections were analyzed under an optical microscope. Muscle samples were reduced to cylindrical fragments, preserving the muscle belly, wrapped in surgical talc, and immersed in liquid nitrogen. They were then included with an Optimal Critical Temperature Tissue-Tek^®^ adhesive (O.C.T., Sakura Finetek, Torrance, USA). The muscles were kept in a freezer at -80 °C until histological sections ten micrometers thick were obtained in a cryostat (Model CM 1850, Leica Microsystems^®^, Wetzlar, Germany) at -20 °C, which were stained in hematoxylin and eosin (HE).

The morphometry of the distal BBFN region was performed with the measurement of 220 nerve and muscle fibers, of all samples of each group, using a microcomputer coupled to a microscope with digital photography (Olympus^®^ BX50, Tokyo, Japan), using software of image capture and analysis (Image Pro-Plus^®^ 6.2 - Media Cybernetics, Bethesda, USA).

The morphometric measurements performed on the distal stump of the BBFN were: the area of nerve fibers, area of axons, minimum diameter of nerve fibers, minimum diameter of axons, myelin sheath area, and myelin sheath thickness [30, 31, 50]. In the muscles, the cross-sectional area of muscle fibers was measured [33, 53, 55].

### Statistical analysis

The measurements taken were organized in spreadsheets and tables in an Excel file (Microsoft Office Excel^®^, Redmond, WA, United States) with the means and standard deviation, which were subsequently submitted to statistical tests. We used the two-way variance test (ANOVA) and then Tukey's test for multiple comparisons between means. Statistical analysis and graphs were performed using the GraphPad Prism version 8.0 program (GraphPad^®^ Software, La Jolla, USA). The level of statistical significance was set at p < 0.05 for all analyses.

## Results

### Functional analysis of rat vibrissae movements

**Figure 2.**
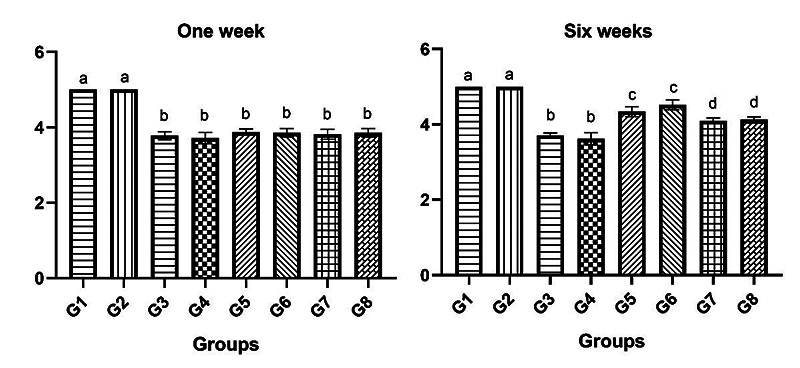
The functional results of the movements of the vibrissae, at 1 and 6 weeks postoperatively, demonstrated with a mean and standard deviation column graph. The different letters (a ≠ b ≠ c ≠ d) indicate a statistically significant difference (one-way ANOVA and Tukey, p < 0.05). **G1:** Control group, right BBFN (without injury); **G2:** Control group, left BBFN (without injury + PBM); **G3:** Denervated right BBFN (neurotmesis); **G4:** Denervated left BBFN (neurotmesis + PBM); **G5:** Immediate repair of right BBFN (neurotmesis + HFB); **G6:** Immediate repair of left BBFN (neurotmesis + HFB + PBM); **G7:** Delayed repair of right BBFN (neurotmesis + HFB); **G8:** Delayed repair of left BBFN (neurotmesis + HFB + PBM).

G1 and G2 groups were used as a reference standard in relation to the normal position and movement of the vibrissae (score 5 for all animals), according to the parameters established by de Faria et al. [54]. In the first week after the experimental procedures, we found similarities between the groups in which neurotmesis occurred (G3-G8), as well as similarities between the control groups (G1 and G2). In the sixth week after surgery, groups G5 and G6 were similar (4.34 ± 0.13 and 4.52 ± 0.13, respectively), with the highest mean for the group that received photobiomodulation. The G7 and G8 groups were similar, with a mean score of 4.10 ± 0.07 and 4.12 ± 0.08 respectively. These results are equivalent to normality results, according to de Faria et al. [54], which considers that values greater than four indicate normal motor function. The denervated groups (G3 and G4) obtained mean scores of 3.70 ± 0.07 and 3.62 ± 0.16, respectively (Figure 2).

### Nerve analysis

**Figure 3.**
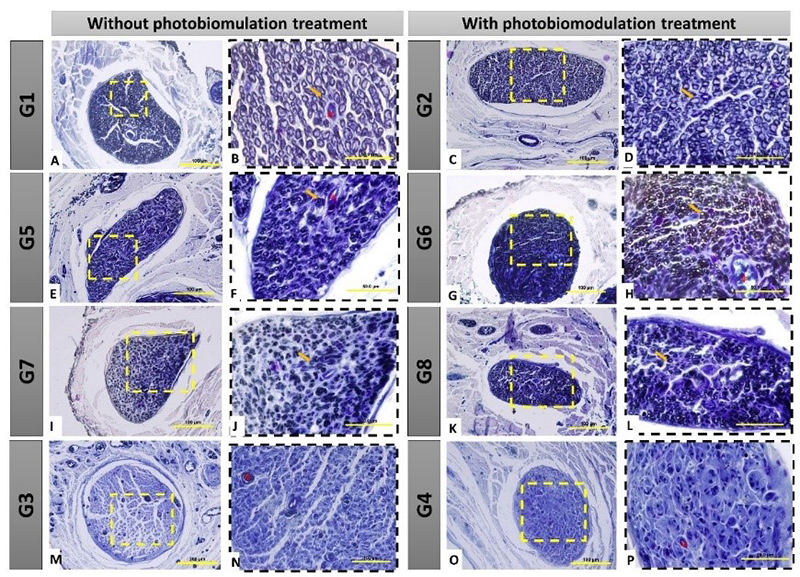
Histological view of the distal stump of the buccal branch of the facial nerve (BBFN) in cross-section, demonstrating the morphology in the different groups, with or without treatment with photobiomodulation (PBM). In 3A-3D, seen at different magnifications, 40x (100 µm bar) and 100x (200 µm bar), myelin fibers with fascicular organization are observed. In 3E-3H, seen at different magnifications (40 and 100x), it was observed that the groups with denervation and immediate repair with HFB also had myelin fibers, but with an apparent smaller size and fascicular organization. In 3I-3L, seen at different magnifications (40 and 100x), it was observed that the groups with denervation and delayed repair with HFB contained myelin fibers of apparent smaller diameter and greater fascicular disorganization. In 3M-3P, seen at different magnifications of 40x (100 µm bar) and 100x (200 µm bar), the groups that underwent denervation and no surgical intervention was performed for repair, demonstrated severe morphological changes in the distal stump of the nerve, resulting from this process, where it was possible to observe the absence of myelin fibers, as well as a large invasion of scar tissue. **G1:** Control group, right BBFN (without injury); **G2:** Control group, left BBFN (without injury + PBM); **G3:** Denervated right BBFN (neurotmesis); **G4:** Denervated left BBFN (neurotmesis + PBM); **G5:** Immediate repair of right BBFN (neurotmesis + HFB); **G6:** Immediate repair of left BBFN (neurotmesis + HFB + PBM); **G7:** Delayed repair of right BBFN (neurotmesis + HFB); **G8:** Delayed repair of left BBFN (neurotmesis + HFB + PBM). Yellow arrow = myelin fiber; Asterisk (*) = blood vessel.

Nerve fibers with larger diameter, myelinated, and with great organization of fascicles, were observed in groups G1 and G2 (Figure 3 A  -3D). In G3 and G4, there was nerve degeneration caused by denervation, not being possible to observe myelin fibers, confirming the negative pattern. Therefore, it was not possible to perform the histomorphometric analysis of these groups (Figure 3 M  -3P).

In groups G5 and G6, in which immediate repair was performed with HFB, we observed invasion of dense connective tissue in the connective coverings, irregular axonal fibers, and apparently smaller diameter compared to the control groups (G1 and G2), however showing sprouting of nerve fibers undergoing regeneration (Figure 3 E  -3H). Therefore, in groups G7 and G8, in which delayed repair was performed with HFB, we found visibly smaller myelin fibers, with disorganization of the architecture of connective coverings and invasion of connective tissue.

**Figure 4.**
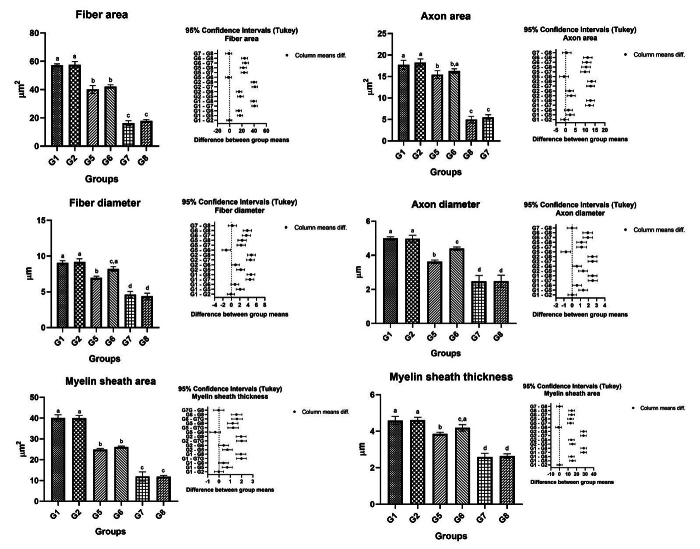
Histomorphometric results of the BBFN of all studied groups demonstrated with the mean and standard deviation in column charts and standard deviation with confidence intervals (Tukey). The different letters (a ≠ b ≠ c ≠ d) indicate a statistically significant difference (one-way ANOVA and Tukey, p < 0.05). **G1:** Control group, right BBFN (without injury); **G2:** Control group, left BBFN (without injury + PBM); **G3:** Denervated right BBFN (neurotmesis); **G4:** Denervated left BBFN (neurotmesis + PBM); **G5:** Immediate repair of right BBFN (neurotmesis + HFB); **G6:** Immediate repair of left BBFN (neurotmesis + HFB + PBM); **G7:** Delayed repair of right BBFN (neurotmesis + HFB); **G8:** Delayed repair of left BBFN (neurotmesis + HFB + PBM).

In the histological and quantitative analysis of the distal stump of the BBFN, it was observed in the analysis of the nerve fiber, axon, and myelin sheath areas, a significant difference between groups G1 and G2 (controls) and the groups that underwent immediate and delayed repair from BBFN; however, the control groups (G1 and G2) were similar to the experimental group G6. We observed that there was no significant difference between the experimental groups in the same repair periods. When comparing the immediate and delayed repair groups, there were higher averages in the immediate repair group. Details of the mean and standard deviation values can be seen in Figure 4 and Table 2.

When we analyzed the nerve fiber, the axon, and the myelin sheath in their diameter, the groups without injury (G1 and G2) were similar to each other, as well as the groups with delayed repair (G7 and G8). However, we observed a significant difference between the G5 and G6 groups, with the highest averages for the group that received photobiomodulation (G6), also noting the absence of a significant difference between G6 and the control groups G1 and G2, in terms of nerve fiber diameter and the myelin sheath diameter (Figure 4 and Table 2).

**Table 2.** Measurements performed on the distal stump of the BBFN.

Groups	Fiber area (µm^2^)	Axon area (µm^2^)	Fiber diameter (µm)	Axon diameter (µm)	Myelin sheath area (µm^2^)	Myelin sheath thickness (µm)
G1	57.14 ± 0.99^a^	17.69 ± 1.08^a^	9.06 ± 0.30^a^	4.98 ± 0.09^a^	40.15 ± 1.50^a^	4.16 ± 0.24^a^
G2	57.61 ± 2.88^a^	18.26 ± 0.82^a^	9.18 ± 0.39^b^	4.96 ± 0.20^a^	39.99 ± 1.29^a^	4.49 ± 0.42^a^
G5	40.08 ± 2.81^b^	15.43 ± 0.91^b^	6.96 ± 0.23^c^	3.62 ± 0.10^b^	24.91 ± 0.54^b^	3.73 ± 0,08^b^
G6	42.15 ± 1.23^b^	16.25 ± 0.54^b,a^	8.19 ± 0.33^d,a^	4.38 ± 0.20^c^	26.14 ± 0.46^c,a^	3.95 ± 0.09^c,a^
G7	16.26 ± 1.67^c^	5.50 ± 0.58^c^	4.61 ± 0.43^e^	2.46 ± 0.34^d^	11.96 ± 2.20^d^	2,4 ± 0,22^d^
G8	17.75 ± 1.13^c^	5.01 ± 0.70^c^	4.38 ± 0.43^e^	2.48 ± 0.35^d^	11.87 ± 0.62^d^	1,91 ± 0,18^d^

Histomorphometric results of the BBFN of all groups studied demonstrated the mean and standard deviation. We present the nerve fiber, axon, and myelin sheath areas and the nerve fiber, axon, and myelin sheath diameters. The different letters indicate a statistically significant difference (one-way ANOVA and Tukey, p < 0.05). G1: Control group, right BBFN (without injury); G2: Control group, left BBFN (without injury + PBM); G3: Denervated right BBFN (neurotmesis); G4: Denervated left BBFN (neurotmesis + PBM); G5: Immediate repair of right BBFN (neurotmesis + HFB); G6: Immediate repair of left BBFN (neurotmesis + HFB + PBM); G7: Delayed repair of right BBFN (neurotmesis + HFB); G8: Delayed repair of left BBFN (neurotmesis + HFB + PBM).

### Muscle analysis

**Figure 5.**
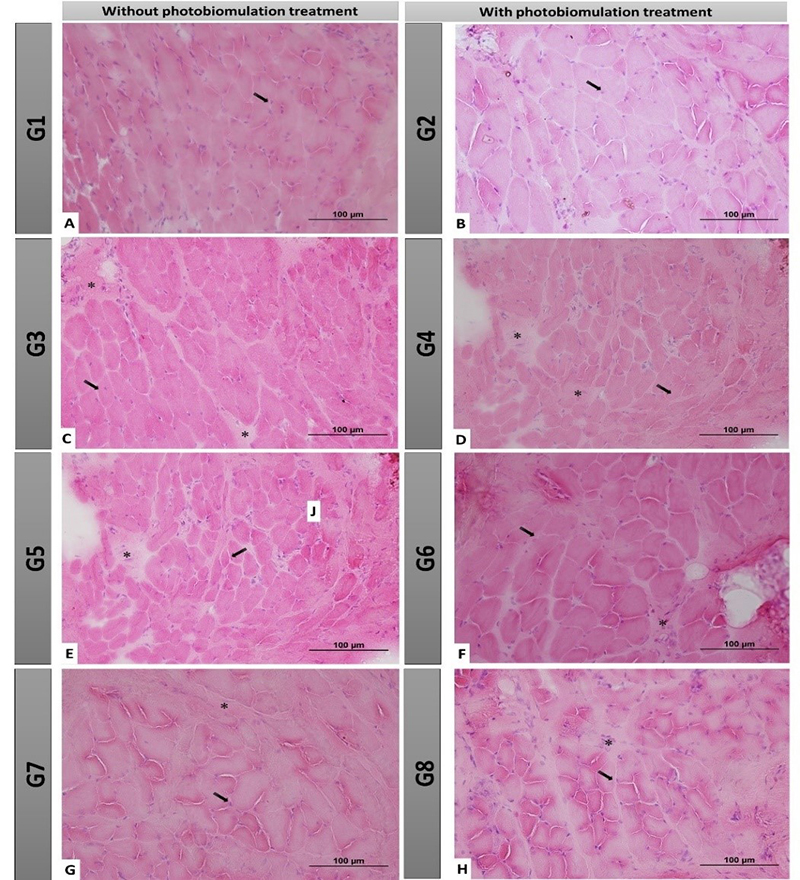
Histological view of the facial muscles in cross-section demonstrating the morphology of the different groups, with or without treatment with photobiomodulation. In Figures 5A and 5B (40x, 100 µm bar), we observe polygonal muscle fibers, with peripheral nuclei and fascicular organization. In figures 5C and 5D (40x, 100 µm bar), the groups that underwent denervation and no surgical intervention was performed to repair the nerve, demonstrated some morphological alterations resulting from this situation, with an apparent dimensional reduction of the muscle fibers and invasion of the connective tissue. In Figures 5E and 5F (40x, 100 µm bar), the groups that underwent denervation and immediate repair with HFB, demonstrated a good histological pattern with few morphological changes. In Figures 5G and 5H (40x, 100 µm bar), we observe the groups that underwent denervation and delayed repair with HFB, demonstrating a good histological pattern, very close to the group with immediate repair. **G1:** Control group, right BBFN (without injury); **G2:** Control group, left BBFN (without injury + PBM); **G3:** Denervated right BBFN (neurotmesis); **G4:** Denervated left BBFN (neurotmesis + PBM); **G5:** Immediate repair of right BBFN (neurotmesis + HFB); **G6:** Immediate repair of left BBFN (neurotmesis + HFB + PBM); **G7:** Delayed repair of right BBFN (neurotmesis + HFB); **G8:** Delayed repair of left BBFN (neurotmesis + HFB + PBM). Black arrow = muscle cell nucleus; Asterisk (*) intramuscular connective tissue.

In qualitative analysis, we observed regular muscle fibers and the organization of connective coverings in groups G1 and G2, demonstrating the morphology of healthy muscles (Figures 5 A  and 5B). In groups G3 and G4, we observed changes resulting from denervation, such as a decrease in the size of muscle fibers and invasion of connective tissue (Figure 5 C and 5D).

In groups G5 and G6, we observed a slight invasion of connective tissue and muscle fibers with a reduced cross-sectional area; however, permeated by muscle fibers with a larger cross-sectional area, the latter being the most common characteristic in the G6 group (Figure 4 E and 4F). Finally, in G7 and G8 we observed a similarity of the qualitative pattern in relation to the G5 and G6 groups, however with apparently smaller muscle fibers (Figure 5 G  and 5H).

**Figure 6.**
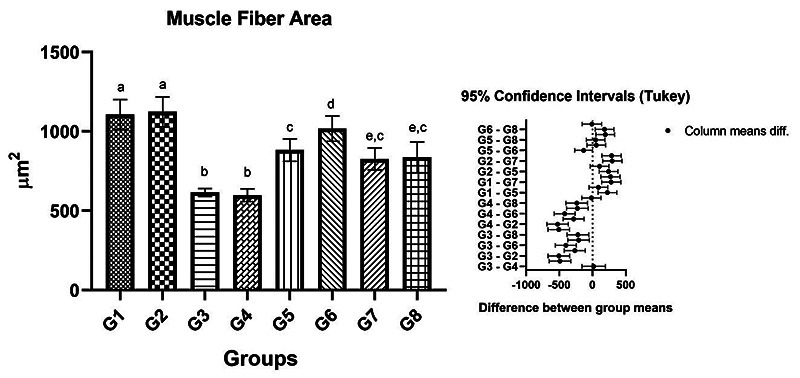
Mean and standard deviation of results of a cross-section of muscle fibers and the difference between groups demonstrated with mean and standard deviation column graph and standard deviation with the confidence intervals. The different letters (a ≠ b ≠ c ≠ d ≠ e) indicate a statistically significant difference (one-way ANOVA and Tukey, p < 0.05). **G1:** Control group, right BBFN (without injury); **G2:** Control group, left BBFN (without injury + PBM); **G3:** Denervated right BBFN (neurotmesis); **G4:** Denervated left BBFN (neurotmesis + PBM); **G5:** Immediate repair of right BBFN (neurotmesis + HFB); **G6:** Immediate repair of left BBFN (neurotmesis + HFB + PBM); **G7:** Delayed repair of right BBFN (neurotmesis + HFB); **G8:** Delayed repair of left BBFN (neurotmesis + HFB + PBM).

In the quantitative analysis of the area of facial muscle fibers, we observed the statistical similarity between groups G1 and G2, (control groups). However, G3 and G4 had the lowest averages but without significant differences between them. In the experimental groups where immediate repair was performed (G5 and G6) a significant difference was observed between them, with the highest mean for the group that received photobiomodulation (G6). The groups referring to delayed repair (G7 and G8) were similar to each other. Furthermore, the delayed repair groups were similar to the immediate repair group G5, but which did not receive photobiomodulation (Table 3 and Figure 6).

**Table 3.** Mean and standard deviation of histomorphometric analysis of facial muscle fiber area.

Groups	Muscle fiber area (µm^2^)
G1	1107.00 ± 94.51^a^
G2	1124.00 ± 94.35^a^
G3	616.10 ± 25.09^b^
G4	598.01 ± 39.58^b^
G5	882.08 ± 70.51^c^
G6	1018.00 ± 79.34^d^
G7	826.04 ± 69.90^e,c^
G8	836.7 ± 96.44^e,c^

The different letters (a ≠ b ≠ c ≠ d ≠ e) indicate a statistically significant difference (one-way ANOVA and Tukey, p < 0.05). G1: Control group, right BBFN (without injury); G2: Control group, left BBFN (without injury + PBM); G3: Denervated right BBFN (neurotmesis); G4: Denervated left BBFN (neurotmesis + PBM); G5: Immediate repair of right BBFN (neurotmesis + HFB); G6: Immediate repair of left BBFN (neurotmesis + HFB + PBM); G7: Delayed repair of right BBFN (neurotmesis + HFB); G8: Delayed repair of left BBFN (neurotmesis + HFB + PBM).

In a general context, the morphological and morphometric analysis of the BBFN showed that nerve regeneration occurred in the groups that received repair with HFB (immediate and delayed), demonstrating myelin fibers with aspects of regeneration and fascicular disorganization. In muscle analysis, we observed the consequences of denervation; however, in the groups with repair, the histological and morphometric results were better compared to the denervated groups. PBM improved the nerve repair process significantly in the immediate repair group. The denervated groups showed total degeneration of the distal stump of the BBFN, greater muscle atrophy, and worse functional outcomes.

## Discussion

Despite advances in tissue bioengineering and surgical techniques, the repair of peripheral nerves is still a challenge for regenerative medicine in the ongoing search for better treatments. Thus, the waiting time for late repair is one of the main factors for unsatisfactory results; therefore, other surgical and non-invasive methods have been tested. In this study, we analyzed the possibility of delayed regeneration of a nerve injury (neurotmesis) of the buccal branch of the facial nerve using, in an unprecedented way, the heterologous fibrin biopolymer as a means of adhesion to the nerve stumps and the effectiveness of a PBM protocol using a low-level laser. We verified that it is possible to use HFB as a means of coaptation in the delayed repair of BBNF. However, we observed that PBM did not positively influence the morphological aspects of the nerve and muscle studied in delayed repair.

The use of fibrin biopolymer as a means of coaptation of nerve stumps has been used by our group of researchers with favorable results [50], demonstrating similar results in comparison with suture thread, considered the gold standard technique [30, 31, 56]. This is also a justification for not using a group with the traditional suture thread. What we tested for the first time was the use of HFB in delayed repairs, obtaining encouraging results. An important aspect of HFB in relation to other sealants available for commercial sale is the low cost, biocompatibility, and impossibility of infections, as it is not made of human blood, unlike other sealants used in clinical practice [57-59].

The post-trauma period for nerve repair of a neurotmesis has been studied. It is generally suggested that immediate repair is the best choice; however, there are still controversial results. In the present study, immediate repair with HFB achieved the best results compared to delayed repair. In a study with pigs, it was suggested that the repair of the facial nerve should be done immediately, and if this is not possible, the repair should be done after seven days and up to 60 days, maintaining satisfactory results but not obtaining satisfactory functional results [60]. It is known in the literature that the period in which the Schwann cells of the proximal stump of the injured nerve remain without contact with the axons of the distal stump during the denervation period impairs the functional results of the peripheral nerves [24, 39, 61].

An important focal point, however little explored in studies that compare delayed and immediate repairs, is the use of photobiomodulation in rehabilitation. We observed that PBM obtained significant results in immediate repair. In the delayed repair at two weeks, PBM did not show statistically superior results, suggesting that the positive influence of the therapy can be better observed in the immediate repair. The benefits of laser therapy (LLLT) in nerve repair are already established, improving the local microenvironment and cellular metabolism for regeneration, leading to better morphometric and functional results [45, 62-64]. Despite this, there is still no consensus on the most efficient protocols for each type of nerve, location, and extent of the lesion [30, 31, 50]. Allied with this, the use of delayed repairs also presents scarce studies.

A possible justification for the lower influence of photobiomodulation on delayed repair may be due to the fact that, after two weeks of injury, Wallerian degeneration was already complete in the distal stump, and the local benefits that PBM generates by improving the morphological aspects of the nerve in repair immediate, it does not occur in the same intensity in the delayed repair. The PBM protocol in the groups with delayed reinnervation (G7 and G8) was performed only after the delayed reinnervation, as although unlikely, PMB could help spontaneous reinnervation of the nerve and greater difficulty in end-to-end neurorrhaphy after two weeks. Therefore, a current disadvantage in performing delayed repair is that the adjuvant method such as low-intensity laser, already consolidated as a means of improving the quality and speed of nerve repair, seems to have little influence in these cases. However, it is necessary to remember that more studies are needed on delayed repair with protocols presenting more PMB sessions, and other doses in order to clarify its possible benefits, which perhaps the protocol of this study was suboptimal. Another fact that we did not study would be that PBM could collaborate in reducing post-traumatic pain.

In delayed repairs, both nerve stumps show inevitable contraction. In studies with sutures for gap correction, tension is inevitable, which reduces the blood supply to the nerve [60]. In the present study with the fibrin biopolymer, this situation can be mitigated by the lower tension in the nerve stumps [29], where they are only approximated for the adhesion of the ruptured fragments.

Regarding the facial muscles, also called mimic muscles, we observed less atrophy in the groups with delayed repair compared to the denervated, and similarly with the immediate repair that did not receive photobiomodulation. Maintaining viable musculature in total nerve section injuries is one of the greatest challenges in regenerative medicine [65-67], being one of the main pillars of maintaining favorable functional results [24]. Our results demonstrated that the repair was able to maintain the transverse dimension of the muscle fibers, thereby helping to improve the functional results of the vibrissae.

Although BBFN is important for innervating rat vibrissae, it is not the only one that performs this function; therefore, the paralysis is not complete with its disruption [68, 69]. However, BBFN denervation causes a significant functional loss, as observed in our results. In animals with delayed repair, despite the time without innervation, they presented better post-repair functional results, leading to a score compatible with the normal movement of the vibrissae, according to the methodology adopted and based on the research by de Faria et al. [54]. Therefore, the final euthanasia of the study was six weeks after the start of the study for all groups, as in the functional study, the literature shows, with several studies, a good functional response in this period [31, 50, 53, 70-72]. Therefore, groups G7 and G8, four weeks after the second intervention (delayed repair after two weeks of neurotmesis) were also sacrificed to maintain the standard in functional analyses, demonstrating a real scenario of the negative effects of delayed repair.

Furthermore, this experimental model using damage to the buccal branch of the bilateral facial nerve is well tolerated by the animal, as it does not completely deteriorate the animal's motor functions, for the reasons previously mentioned. Other similar studies using this methodology have already been published, demonstrating safe driving for animals and reporting no discomfort to animals [30, 31, 50, 73-75]. However, caution must be taken if the study is conducted with damage to the facial nerve trunk, as it will result in total paralysis of the animal's face. However, a limitation must be considered of this bilateral technique when using PMB in association with treatment, because experimental studies in rats have shown positive systemic effects with the use of PMB [76-78]. However, in these studies, the PMB is performed in the animals' caudal veins/arteries, unlike our study, in which we performed the lesion locally along the BBFN path.

Therefore, briefly contextualizing this in vivo preclinical study, when we compare it with a previous study by our group of researchers with a similar design, but without evaluating the effects of delayed repair [51], a future perspective that has been reported, we observe the deleterious effects of this delayed repair, but which is often necessary for the clinical stabilization of the patient for subsequent recomposition of injured nerves.

In future studies, it becomes interesting to use new photobiomodulation protocols with a greater number of sessions and with different doses, analyzing the late repair, or the use of LED (Light-Emitting Diode), in the search for a better understanding of the mechanisms of photobiomodulation in lesions with a longer waiting time for repair, which is common in clinical rehabilitative practice. The use of different protocols, with irradiance different from that used in our study, may perhaps improve peripheral nerve repair in both situations (immediate and delayed) and may be considered as a possible limitation of the study.

## Conclusions

We investigated, in an unprecedented way, the effects of the use of heterologous fibrin biopolymer (HFB) and photobiomodulation (PBM), on delayed nerve repair of the buccal branch of the facial nerve (BBFN), investigating the morphological and functional effects. We demonstrated, with the results obtained, that HFB was effective for repairing BBFN in cases where the repair had a waiting period for reconstructive surgery, allowing axonal growth in the distal stump, and minimizing muscle atrophy, which allowed better functional results. We also observed that PBM with the protocol used had a positive influence on immediate repair; however, without significant influence on delayed repair. Therefore, the use of this bioproduct (HFB) in delayed repairs, which are common in several clinical situations in peripheral nerve injuries, is effective, generating prospects for clinical studies in regenerative medicine.
